# Health, human rights and mobilization of resources for health

**DOI:** 10.1186/1472-698X-4-4

**Published:** 2004-10-08

**Authors:** Reidar K Lie

**Affiliations:** 1 Department of Clinical Bioethics, National Institutes of Health, Bethesda, MD 20892, USA; 2 Department of Philosophy, University of Bergen, Sydnesplassen 7, N-5007 Bergen, Norway

## Abstract

**Background:**

There has been an increased interest in the role of a human rights framework to mobilize resources for health.

**Discussion:**

This paper argues that the human rights framework does provide us with an appropriate understanding of what values should guide a nation's health policy, and a potentially powerful means of moving the health agenda forward. It also, however, argues that appeals to human rights may not necessarily be effective at mobilizing resources for *specific *health problems one might want to do something about. Specifically, it is not possible to argue that a particular allocation of scarce health care resources should be changed to a different allocation, benefiting other groups. Lack of access to health care services by some people only shows that something has to be done, but not what should be done.

**Summary:**

The somewhat weak claim identified above together with the obligation to realize progressively a right to health can be used to mobilize resources for health.

## Background

During the past few years there has been an increasing interest in attempts to utilize a human rights framework to argue that we have obligations, in one way or another, to do something about the human suffering among the large number of poor in the world. The argument is that the suffering of the poor is a violation of their human rights, and the international human rights instruments place an obligation on us to do something about it. The exact details of the argument vary among different commentators, but they all have in common this basic argument structure. Rarely, however, are we provided with any details about exactly how one should understand particular violations of human rights or exactly how one arrives at recommending a particular action to rectify the alleged violation of a human right. Nevertheless, many are confident that a linkage to human rights will prove useful when we want to mobilize resources for the world's poor. In the words of Paul Farmer

Of course, it is easy to demand more resources; what is hard is to produce them. But if social and economic rights are acknowledged as such, then foundations, governments, businesses, and international financial institutions-many of them awash in resources-may be called on to prioritize human rights endeavors that reflect the paradigm shift advocated here [1], p. 244.

In this paper I want to examine this claim as it relates to health: How can one use a human rights framework to mobilize resources for health? If we want to use this framework presumably we can make demands on the basis of two types of reasons. Those who lack access to resources can argue that the total amount available to promote health should be increased, or they can argue that the allocation of available resources should be made in a different way, giving them access to health care services, but denying other, less deserving groups access. One may, of course, also wish to make both demands. In this paper I shall argue that the human rights framework does provide us with an appropriate understanding of what values should guide a nation's health policy, and a potentially powerful means of moving the health agenda forward. I shall, however, also argue that appeals to human rights may not necessarily be effective at mobilizing resources for *specific *health problems one might want to do something about.

## Discussion

### The international human rights framework

One may understand the claim that "health is a human right" in at least three different ways. First, we may simply want to say that health is important, that we should all do what we can to promote health, and we may even expect that referring to health as a human right might produce an emotional response in our audience, motivating them to action. If this is what we want to do, and if claiming that "health is a human right' does in fact produce this type of response, then utilizing a rights framework can indeed be expected to mobilize resources. While it is undoubtedly true that this strategy will sometimes be effective, its effectiveness will more often than not depend on the immediate reaction to the deprivation of a particular group, and that group's ability to elicit sympathy for their cause, rather than a legitimate policy response where all competing claims have been taken into account. Second, we may want to make a moral claim of a particular type. There is an extensive philosophical discussion of how one should understand the concept of "a right" in general, and a "right to health" in particular. While not denying the importance of this discussion, it does not have much relevance to the problem addressed in this paper: does one have a reason to believe that the claim that "health is a human right" will mobilize resources for health? Even if we take the strongest claims to be true, for example that there are moral obligations on each one of us to do something quite specific to improve other people's health, we still have to provide an account of how to translate these moral obligations into effective action. That is why I want to limit my discussion to an understanding of "health as a human right" as those specific legal obligations on states that arise out of international law. International law in this context refers primarily to The Covenant on Social, Economic and Cultural Rights, but also the General Comment by the UN Committee on Economic, Social and Cultural Right on article 12 (on the right to health) of the International Covenant on Economic, Social and Cultural Rights, and the first report of the recently appointed Special Rapporteur with a mandate to focus on the right to health [2,3]. In addition, there are a number of cases that have been decided on the basis of a right to health. These cases are particularly important when we attempt to understand what is meant by a "right to health" in international law. If we take a right to health in this third sense, we have at least in principle identified a way of understanding rights that can lead to an effective mobilization of resources.

The issue to be addressed is therefore: Can one on the basis of international law argue that a person's right to health is violated if that person is denied access to health care services or the underlying determinants of health on economic grounds; that is, either because that person does not have sufficient resources herself to pay for the services or the state claims that it does not have sufficient resources to pay for the necessary services. If one could establish that such cases are violations of international law, then one would have an effective way of mobilizing resources for health based on a human rights framework.

If one adopts this approach, one challenge is, in the words of the Special Rapporteur, that "although there is a growing national and international jurisprudence on the right to health, the legal content of the right is not yet well established (#39)". Some indication of how one should understand the right to health in international law is nevertheless provided in the key, authoritative documents and in the court cases that have been decided on the basis of right to health challenges. Some of the statements made in the General Comment might indeed lead one to believe that a state has strong legal obligations to provide sufficient resources to ensure adequate health for all. It is said, for example, that "health facilities, goods and services must be affordable for all. Payment for health care services, as well as services related to the underlying determinants of health, have to be based on the principle of equity, ensuring that these services, whether privately or publicly provided, are affordable for all, including socially disadvantaged groups (12 1 iii). One could take this to mean that it prohibits denial of health care services on economic grounds. Other statements seem to support this claim " [F]unctioning public health and health care facilities, goods and services, as well as programmes, have to available in sufficient quantity within the State party (12(1)). Central to the General Comment is the principle of non-discrimination: "...the Covenant [on Economic, Social and Cultural Rights] proscribes any discrimination in access to health care and underlying determinants of health ... on the grounds of race, colour, .... health status ... and civil, political, social or other status" (18). This is reinforced by the special rapporteur: "Accordingly, international human rights law proscribes any discrimination in access to health care, and the underlying determinants of health, on the internationally prohibited grounds, including health status, which has the intention or effect of impairing the equal enjoyment of the right to health" (26).

Taken together, these statements seem to give a strong endorsement to the claim that it is a violation of a person's health rights to deny him treatment on the grounds that treating that person is too expensive for the state. If one denied treatment to persons who happen to have diseases that are expensive to treat, one does indeed discriminate on the basis of "health status", one of the prohibited grounds, and one does not ensure equal access to health care services to all.

There is, however, one important qualification to this claim. Although the state has an obligation to provide health care in "sufficient quantity", "the precise nature of the facilities, goods and services will vary according to numerous factors, including the State party's developmental level" (12 1). Furthermore, "the Covenant provides for progressive realization and acknowledges the constraints due to the limits of available resources" (30). It is, therefore, in spite of the strong statements that everyone should be assured access to health care, in principle legitimate for a state to claim that it can deny access to health care to patient groups who happen to have diseases that are expensive to treat.

A state's claim that it does not have sufficient resources to provide access to health care or its determinants to a particular group because of its costs can, of course, be challenged. We would then need some principled way of adjudicating between the competing claims: on the one hand those of the group denied health care access claiming that its health rights are violated and on the other hand those of state claiming that its resources can be utilized better elsewhere. The official documents on how we should understand a right to health do not provide us with much guidance on how one should adjudicate between such competing claims. As we shall see in the next section, neither apparently do the cases which have been brought forward as violations of a right to health.

### The lack of a principled way of adjudicating between competing claims

The two recent South African cases provide a particular striking example of the challenge of using a reference of a right to health or the courts to mobilize resources for health. The South African constitution, article 27 (1) gives everyone a right to have access to health care services, including reproductive health care and in article 27 (2) it says that the state must take reasonable legislative and other measures, within its available resources, to achieve the progressive realization of these rights. These national provisions, of course, reflect the ones in the UN Covenant. In November 1997, the Constitutional Court of South Africa decided on a case involving the scope of such a "right to health" [4]. The case involved a diabetic man with ischaemic heart disease and cerebro-vascular disease with chronic kidney failure. He was rejected for the dialysis program on the grounds that there was a shortage of dialysis machines and dialysis had to be reserved for people with acute renal failure or for patients who are candidates for kidney transplantation. He appealed this decision to the Constitutional Court on the grounds that the South African Constitution gives every person a right to life and that nobody may be denied emergency medical treatment. The Court rejected the appeal on the grounds that this was not an emergency treatment, and that a right to life should be interpreted as a right to non-interference, but not necessarily a duty to sustain life. In the words of the Court

"It [article 27(3)] provides reassurance to all members of society that accident and emergency departments will be available to deal with unforeseeable catastrophes which could befall any person, anywhere and at any time" (section 51)

and

The applicant suffers from chronic renal failure. To be kept alive by dialysis he would require such treatment two to three times a week. This is not an emergency which calls for immediate remedial treatment. It is an ongoing state of affairs resulting from a deterioration of the applicant's renal function which is incurable" (section 21)

Although the appellant did not appeal the decision on the basis of the South African Constitution's article 27 (1) giving everyone a right to have access to health services, the Court did discuss this matter. The Court pointed out that it is not disputed that the Department of Health

"does not have sufficient funds to cover the cost of the services which are being provided to the public ... This is a nation-wide problem and resources are stretched in all renal clinics throughout the land. Guidelines have therefore been established to assist the persons working in these clinics to make the agonising choices which have to be made in deciding who should receive treatment and who not" (section 24).

The Court further maintained that the current guidelines are justified by the fact that more patients would benefit from the limited resources available than by any alternative use of resources, and that "it has not been suggested that these guidelines are unreasonable or that they were not applied fairly and rationally when the decision was taken by the Addington Hospital that the appellant did not qualify for dialysis" (section 25).

The second case involves use of nevirapine to prevent perinatal HIV transmission[5,6]. As is well know, the South African government has until recently refused or been reluctant to provide antiretroviral treatment for HIV, both for HIV positive people and to pregnant women to prevent perinatal HIV transmission. The South African Treatment Action Campaign brought a suit against the government that the policy regarding the use of nevirapine violated the constitutional right to health. The Supreme Court ruled in 2002 with the Treatment Action Campaign, affirming that the government's policy violated the Constitution's right to health. It is important, however, to note the basis for this ruling.

The South African government had claimed that providing nevirapine to pregnant women would be too costly in terms of infrastructure, in particular provision of testing and counseling, and that its safety and efficacy had not been sufficiently demonstrated in a South African context. The Supreme Court disagreed with both of these claims. The issue was "whether it was reasonable to exclude the use of nevirapine for the treatment of mother-to-child transmission at those public hospitals and clinics where testing and counseling are available". Regarding the safety and efficacy issue, the court cited scientific opinions which made these claims completely unreasonable.

The Court did not address the issue of an appropriate allocation of resources. Accepting the Court's decision would not require the South African government to allocate additional resources to health care delivery nor would it require it to re-allocate existing resources for health:

A potentially lifesaving drug was on offer and where testing and counseling facilities were available it could have been administered within the available resources of the state without any known harm to mother and child.

In fact, the Court, as in the dialysis case, rejected any role for the courts in resource allocation decisions, instead adopting a criterion of evaluating government policies on the basis of a criterion of "reasonableness", from a previous right to housing case. In this previous case, the court would not decide "whether other more desirable or favourable measures could have been adopted, or whether public money could have been better spent"[7] (41). A policy is reasonable if it is comprehensive and well coordinated; is balanced, and does not exclude a significant segment of society; and responds to the urgent needs of those in desperate circumstances. In the nevirapine case the Court affirmed that any right did not impose an obligation on the state "to go beyond available resources or to realise these rights immediately" (para 32). The court did concede that it would be reasonable for the government to carry out a research project to determine the safety of the drug before a wider implementation, but that it would not be reasonable to deny people access to the drug outside of research and training sites.

If one therefore follows the views of the South African Court regarding a right to health, it would seem to be difficult to use the courts to challenge a particular allocation of health care resources. The Court felt that the provincial administrations should make decisions as to how funds for health care should be spent and that courts should be "slow to interfere with rational decisions taken in good faith by the political organs and medical authorities whose responsibility it is to deal with these matters" (section 29).

And

"courts are not the proper place to resolve the agonising personal and medical problems that underlie these choices. Important though our review functions are, there are areas where institutional incapacity and appropriate constitutional modesty require us to be especially cautious. Our country's legal system simply cannot replace the more intimate struggle that must be borne by the patient, those caring for the patient, and those who care about the patient. The provisions of the bill of rights should furthermore not be interpreted in a way which results in courts feeling themselves unduly pressurized by the fear of gambling with the lives of claimants into ordering hospitals to furnish the most expensive and improbable procedures, thereby diverting scarce medical resources and prejudicing the claims of others" (section 58).

One might, of course, want to criticize this view of the role of the courts, as has been done by Darrel Moellendorf.[8] Moellendorf argues that the courts should rule on what is meant by "within available resources" and that the Constitutional Court previously has recognized that rulings on socio-economic rights do have budgetary implications: "the Court's role in upholding socio-economic rights is not foreseen as limited to the framework of existing national or provincial budgetary allocations. Rather the court may pass judgments on these rights, as with other rights, that require a change in fiscal priorities" (p. 331). In this paper, however, I shall suggest a different approach that may be more promising if one wants to use an appeal to a right to health to mobilize resources for health.

### The problem of resource allocation

The central issue with regard to differential access to health care services is, on what basis one can claim that a state does not spend a sufficient amount of resources on health care, relative to its general wealth, and on what basis can one claim that a state does not use the resources it devotes to health care appropriately. If a state does not allocate sufficient resources to health care, or uses its available resources inappropriately, a citizen could claim that the state violates her right to health when a particular health care intervention is denied her, and when that intervention would be available to her if the state increased its allocation to health care to an acceptable level, or re-allocated resources within the health care sector to an acceptable mix of interventions.

Focusing attention on this set of questions is important because they are at the heart of debates about health care access, in both resource rich and resource poor settings. We want to know whether denying a person access to a life-saving intervention because it is too expensive for a given health care system is a denial of his right to health. This can be the typical high cost therapies in rich countries, such as bone marrow transplantation, or it can be antiretroviral treatment for HIV in resource poorer countries. If we assume that a state is *in principle *entitled to make judgments about when a particular treatment is too expensive to provide within its healthcare systems, given available resources, we have to find a way to examine claims about appropriateness, and provide criteria for how these judgments are to be made. At least in the two South African cases the Court has been reluctant to get involved in this type of questions.

It should come as no surprise that this is the case. As the ever growing literature on the ethics of health care resource allocation demonstrates, there is no clear consensus about how one should balance the various legitimate concerns and values involved in making these kinds of decisions. Judgments will have to be made, and, unless there are grave violations of due process, or a gross misapplication of the principles of resource allocation, it seems unlikely that one could make a principled criticism of a particular decision that could be used as a basis for a claim against the state. This is probably why the courts have been reluctant to criticize allocation judgments made in good faith by government bodies.

In spite of this largely *negative *conclusion, I shall now nevertheless a strategy that shows that appeals to health as a human right might nevertheless be used to argue that denial of care on economic grounds is a violation of that right.

### Using human rights to mobilize resources for health

The central guiding principle in the international health and human rights documents is that of non-discrimination. It is prohibited to deny a person access to health care or its determinants on the basis of characteristics such as race or religion, but also, as we saw above, on the basis of social and health status: health care services should be accessible to *everyone*, there should be *equal *access to health care, and health care should be *affordable for all*. It is fairly easy, and uncontroversial, to establish that a policy such as the one accepted during apartheid in South Africa, of denying access to health care services on the basis of race, is a violation of a right to health. It is more difficult to agree on how we should understand the principle of non-discrimination on the basis of *social *and *health *status. One might be tempted to conclude that the principle of non-discrimination on the basis of social status implies a right for everyone to access the *same *bundle of health care services. An unqualified acceptance of that claim, however, would obviously be unacceptable: there are going to be health care services which provide very little benefit, but which are hugely expensive, which may be accessible to the very rich, but which should not by any account be included in the bundle of health care services accessible to all, even by the most egalitarian standards of justice. We therefore need *some *account that will specify *which *health care services should be accessible to all, even within a rights approach to health care. In order to give a satisfactory account of that problem, we again need an appropriate value framework for prioritization. It seems to me that there is nevertheless something we can say, even before we have a fully worked out framework for prioritization, if we take a right to health approach. If a range of health care services, known to be effective in significantly preventing premature death or significantly increasing quality of life, is available to a substantial portion of a country's population, but is not available to a particular group of people, such as the very poor, this is a violation of the right to health of the members of that group. The qualifiers indicate that a lot of work needs to be done to give precise content to this claim, but for the purposes of this discussion, this somewhat vague claim will be sufficient. It is easy to identify examples: If antiretroviral treatment for HIV is available to most people in country, but is not available to people in the lowest 1% income bracket, we can conclude that there is a prima facie violation of their health rights.

Although one might agree that such a principle identified here does indeed follow from the international human rights documents, there are some obvious objections if one's aim is to show that an international human rights framework can be effective at mobilizing resources for health. First, if one accepts this principle of non-discrimination based on social status, why should one not accept a principle of non-discrimination based on health status, and argue analogously that if a small group of persons is denied access to health care services because of the illness that they happen to have, at the same time as the majority of the population has access to health care for their illnesses, this is an illegitimate form of discrimination? Second, can not a state use the same principled objection to mobilization of resources in cases of discrimination based on social status, that its limited resources are better used elsewhere? Third, are any real policy alternatives ruled out by this principle, or is it not so general that all types of health policies is compatible with it.

The first criticism says that if we find it objectionable that the poor are treated differently from the rich with regard to health care access, why should we not find it objectionable that those who happen to have late stage breast cancer are treated differently from those who happen to have disease that can be treated more cheaply?

Why could not a person with late stage breast cancer argue successfully that a decision not to fund the known effective treatment she suffers from is of the same type as a decision not to fund treatments the poor suffer from?

One response to this objection would be that decisions regarding what constitutes a non-discriminatory policy are different in these two cases. When deciding whether who to give access to health care services in general, we do not discriminate on the basis of what illnesses people have: we look at other characteristics, such as gender, age, ethnic background, social status, and income level. When deciding what bundle of health care services to provide within a nation's health care system, other considerations are relevant. We would then look at the effect of the various interventions, their costs, how each individual benefits from the intervention, and what the cumulative benefit for the population is. A discriminatory practice in the first type of decision would be to single out a particular income group for preferential treatment, while a discriminatory practice of the second type of decision would be to single out a particular illness for preferential treatment. It would not be discriminatory to distribute resources on the basis of, for example, health effects of the various interventions. The reason why we have more problems in identifying inappropriate discrimination on the basis of health status is that there is less agreement on how we should identify interventions that have the same effect on health status across illness groups.

The second objection is more difficult to answer. Let us assume that there is a country with a government that tries to allocate resources justly. Although the government tries its best, there continues to be lack of access to health care services to the poor. The government has also done its best in raising the resources allocated to health care, but they have still not been able to fund life-saving interventions for a particular group of patients. Because of its generally sound economic policies, the country has experienced more than the expected economic growth over the past few years, and there is a budget surplus. The government now has to decide what to do with the additional money: Should they remove continued barriers of access to the poor, or should they ensure funded for the neglected disease? We assume that the government cannot do both. It seems to me that the principle of non-discrimination within a human rights approach to health care does not provide us with guidance about how we should go about solving this type of problem.

In spite of this problem, there is one answer we can give, albeit a limited one, but still with a potential for identifying some inadmissible policies this imaginary country might want to consider. Within the human rights framework there is a recognition that any given country cannot fulfill its obligations immediately, but there is an obligation of *progressive realization *of a right to health. This places an obligation on the country to do *something *to increase access to health care services during periods of economic growth. On that basis we could justifiably criticize government *inaction*, but we would not be justified in proscribe a particular action.

The third objection is that the principle of non-discrimination is so general that it would not rule out any particular health policy. This is not the case. One currently fashionable proposal is that governments should identify an essential package of health services based on a criterion of cost-effectiveness. This package should be available to everyone, regardless of ability to pay. Those who are wealthy would be able to, or indeed encouraged to, pay for additional health care services using their own funds. WHO has called this proposal "the new universalism". Classic universalism would obligate governments to provide everything medically useful to everybody. How should we evaluate this proposal in terms of a human rights approach to health care delivery?

Let me first examine the arguments in favor of this proposal. First, it is quite clear that the policy is pro-poor, in the sense that it would increase access to health care services for the poor compared with what is currently available to them. Data from many country shows that government subsidies today go disproportionally to the wealthy to pay for interventions of low cost-effectiveness, while the poor suffer from conditions which require interventions of high cost-effectiveness. Requiring that governments fund a package of services of high cost-effectiveness available to everyone would therefore shift government resources from the rich to the poor. Second, according to the principle of non-discrimination within the human rights tradition, governments should not discriminate against a particular group of people in terms of access to health care services. This policy seems to satisfy that requirement. The basic package is accessible to everyone, and the government does not subsidize health interventions for non-poor if they are not also subsidized for the poor, unlike what is the case in many countries today. For these arguments, see [9,10]. The following sentence sums up the rational for this position:

Although many countries cite equity as the reason for strong government controls, public sector-controlled policies do not have a good track record on equity. In Indonesia, for example, the rich receive almost three times as much public health care as the poor. In China in the early 1980s, rural households-almost 80 percent of the population-received just 29 percent of public health spending. In Tanzania the richest fifth of the population use more than twice as many government hospital beds and more than four times as many outpatient services as the poorest fifth. In Côte d'Ivoire less than one-quarter of the rural poor who were sick received any form of medical care, as compared with half of the urban rich. In Peru only 20 percent of the poor received care, versus 57 percent of the rich. In general, when government expenditures are concentrated on urban areas and on hospitals rather than on basic services, the results are highly inequitable, governments are essentially subsidizing the rich. [11]

This conclusion only follows, however, if the principle of government non-discrimination only applies to the proportion of public resources which goes to different population groups. If the non-discrimination principle is limited in this way, and if the public system is available to everyone regardless of ability to pay, a two-tiered system where the wealthy have access to a much broader range of health care services because they are able to pay for the additional services from their own income is not in conflict with a right to health. If, on the other hand, one takes the position that a right to health should be understood in terms of equality of access to health services in general, then an explicit acceptance of at least some types of two-tiered systems is not in accordance with a right to health.

There are a number of considerations which speak in favor of this expanded notion of government responsibility. A government does not only have an obligation to provide or to finance health care services. In fact, in recent discussions about health sector reform, these types of government obligations have been de-emphasized and instead there has been a focus on a government responsibility to regulate, facilitate and ensure that people have access to health care services, whether they are provided or financed by government, non-profit organizations or for profit companies. If one takes this role seriously, focusing only on how government resources are spent is at best only one component of government obligations to fulfill the right to health of their population. Persistent inequalities of access to health care, whether they are caused by imbalances in public resources, or caused by differences in income between population groups, should therefore be regarded as an essential concern of equity and rights oriented policies.

## Summary

I have in this paper argued that appeals to health as a human right are not going to be helpful if we want advice on how we should allocated resources among different patient groups. Many of the most difficult problems of resource allocation are therefore not addressed by a human rights approach. However, I have also argued that some inequalities of access can be criticized: One can say that it is a violation of human rights if one group systematically has less access to health care services compared with other groups. There is, however, not much more on can say; in particular, one cannot proscribe a *particular *corrective to that violation of health as a human right. All one can say is that the state has an obligation to do *something *about the injustice.

This result may not be very satisfactory for those who are concerned about doing something about lack of access to health care. However, if one takes the requirement of progressive realization seriously, together with the periodic reporting requirement, one might conceivably have a powerful basis for criticism of government policies. Governments are obligated to report to the UN, and, as I have shown, there is a basis on which one can criticize them for not fulfilling their health rights, also with regard to their economic policies. This creates an obligation on countries to show that *something *has been done when the next periodic report is done to and they would have to take steps to improve access in *some *respects. While this may seem to be a modest requirement, it can, together with appropriate political pressure, lead to significant, positive changes in health care systems. But it is undeniable that it is much less than what many who advocate a human rights approach to health care access hope can be achieved by utilizing this framework. It is also true that it is generally recognized that the reporting requirements, at least with regard to health, are not very effective. Strengthening the reporting and monitoring mechanisms of the UN system with regard to health should therefore be the highest priority for those who want to emphasize a human rights approach to health sector reform.

## Competing interests and acknowledgments

The opinions expressed are the author's own. They do not reflect any position or policy of the National Institutes of Health, Public Health Service, or Department of Health and Human Services.

## Pre-publication history

The pre-publication history for this paper can be accessed here:


